# Extending repair in peer interaction: A conversation analytic study

**DOI:** 10.3389/fpsyg.2022.926842

**Published:** 2022-08-29

**Authors:** Mia Huimin Chen, Shelly Xueting Ye

**Affiliations:** Department of English, University of Macau, Macau, China

**Keywords:** peer interaction, conversation-for-learning, conversation analysis, learning opportunities, repair

## Abstract

Peer interaction constitutes a focal site for understanding learning orientations and autonomous learning behaviors. Based on 10 h of video-recorded data collected from small-size conversation-for-learning classes, this study, through the lens of Conversation Analysis, analyzes instances in which L2 learners spontaneously exploit learning opportunities from the on-task public talk and make them relevant for private learning in sequential private peer interaction. The analysis of extended negation-for-meaning practices in peer interaction displays how L2 learners orient to public repair for their learning opportunities in an immediate manner and in so doing, how different participation framework is being utilized to maximize their learning outcomes. As these extended repair practices are entirely managed by learners themselves, they yield both efficient and inefficient learning outcomes. Findings reveal that learners frequently resort to their peers to recycle the focal trouble words for learning opportunities, shifting their participating role from the on looking audience to active learners. By reporting the rather under-researched post-repair negotiation-for-meaning sequence in peer interactions, the study highlights the relevance between on-task classroom activities and private learning, contributing to understanding private learning behaviors in the language classroom and learning as a co-constructed activity locally situated in peer interaction.

## Introduction

Constrained by classroom settings and pedagogical designs, conventional teacher-fronted language classes have been criticized for secluding language use from language teaching (Kasper and Kim, 2015). Teaching instructions in teacher-fronted classes are frequently delivered in the form of monolog, IRF (initiation/response/follow-up) and choral repetition (Liu, 2008). Responding to the landmark call for reconceptualizing the second language (L2) learning (Firth and Wagner, 1997), the teaching and learning of L2 are increasingly connected to the real social contexts in which the target language is used (Gardner, 2015). Sequentially, pedagogical arrangements such as conversation-for-learning (Kasper, 2004; Kim, 2012, 2017a,b; Kasper and Kim, 2015; Hauser, 2017a), Talk Time and Language Corner (Gao, 2009) are implemented to complement conventional language teaching, augmenting L2 learners’ exposure to and the employment of the target language. These arrangements offer learners more interactional opportunities and enhance their autonomous learning awareness through maximizing their engagement in simulative real-life interaction (Mori, 2002). The present study reports private learning behaviors in a multiparty conversation-for-learning class. Through the analysis of peer interaction, the paper introduces how L2 learners orient to learning opportunities in private and make use of the possible knowledge asymmetries in peers to maximize individual learning outcomes. The study is mainly informed by three lines of research: classroom discourse; conversation-for-learning (CfL); CA-for-SLA.

A wealth of CA studies has discussed interactional sequences between teachers and students. Given the complexity of classroom discourse, different participation frameworks and interactional trajectories are necessarily distinguished to understand how participants collaboratively construct the orderly classroom activity. The literature on peer interaction in class, however, is relatively sparse. Albeit it has been argued students benefit from interactions with both teachers and peers in the classroom in terms of social and academic development (Chen et al., 2020), there has not appeared “much work that combines fine-grained sequential analyses with analyses of the multiparty design of classroom conversations” (Ahlund and Aronsson, 2015, p. 67). An emerging body of research intends to display the sophisticated designs of different participating frameworks in the classroom by analyzing peer interactions. This vein of research includes how learners retrospectively orient to learning achievements in Content and Language Integrated Classrooms (Jakonen, 2018) and how learners use their L1 to scaffold the off-task peer interactions in EFL class (Stone, 2019).

The research of CfL, upon which the present study builds, has been mainly focused on dyadic interactions between language experts and novices (e. g., Kasper, 2004; Hauser, 2013, 2017a,b). In rare cases, Kim (2017a,b) analyzed interactional practices between two Korean EFL learners and an American language expert, still addressing how learning opportunities are occasionally generated by a language expert for L2 learners. This vein of research has demonstrated how learning opportunities naturally emerge in CfL, and how participants make use of the language and knowledge discrepancy to orient to temporal learning opportunities. In a CfL setting, language becomes the vehicle for communication and the goal of learning. The previous literature has highlighted how language experts scaffold learning for novice learners. Nevertheless, relatively little attention has been given to the participating frameworks in multiparty CfL arrangements and how learners themselves collaboratively assist each other’s learning and sense-making.

Employing Conversation Analysis (CA), this study addresses the issue of “schism” (Sacks et al., 1974) or “schisming” (Egbert, 1997) in the classroom by displaying how on-task public interaction elicits private peer learning activities and how peer talks separate the ongoing classroom interactions. Constructed in the terrain of social interaction, schism refers to the phenomenon that a sole conversation divides into several conversations in a group talk (Sacks et al., 1974). According to Egbert (1997), the transformation from a single conversation to multiple conversations requires sophisticated designs and collaborative efforts. As conversational mechanisms are subject to different designs and uptakes in a multiparty setting, it is significant to understand how interactants mobilize available resources to subdivide a conversation and construct other interactive agendas in minor groups. Although the orderly organization of classroom discourse makes relevant collaborative participation, few studies have discussed the construction of classroom activity regarding schisming and different participation frameworks. To gain an understanding of the construction of classroom discourse, it is significant to consider various designs and participation frameworks in terms of distinct interactional purposes (Coughlan and Duff, 1994).

Based on video-recorded data collected from an interactive English course, the aims of the study are tripartite: 1. to document the relatively under-reported learning opportunities and learning behaviors in multiparty CfL class; 2. to contribute to the understanding of peer interactions, classroom discourse and participation framework in EFL classroom; and 3. to analyze the relevance of post-repair negotiation for meaning (PRNfM) practice for the on-task teaching task and L2 learning. This study highlights the connection between the on-task public activity and the initiation of private peer talk through the close examination of the construction of peer interactions. Especially, it concentrates on private PRNfM sequences that appear upon the completion of a public repair and are unfolded within learners. In line with other research employing learning behavior tracking methodology within the terrain of CA-for-SLA (Markee, 2008), the present study analyzes how L2 learners retrieve key vocabulary in preceding public repair and recycle them in sequential private peer interactions for individual learning opportunities. In so doing, the study showcases how different participation framework is being utilized to maintain the orderly progressivity of classroom discourse and meanwhile, how L2 learners mobilize their agency to learn. Findings of the study suggest the analysis of peer talk is necessary for the understanding of the orderly construction of classroom discourse and the autonomous management of learning opportunities in private.

## Literature review

### Classroom discourse and peer interaction

Teachers and students exchange knowledge in a classroom in two primary ways: direct transmitting knowledge from teachers to learners; conversational approaches (Houen et al., 2018). While conventional teaching activities in the classroom prefers direct transmission of knowledge, communicative class adopting conversational approaches creates adequate interactional space for learners to be active conversational participants (Durden and Dangel, 2008; Houen et al., 2018). As Seedhouse (2015) has pointed out, classroom interaction is a highly adaptive system, requiring complex designs to sustain order. Students’ behaviors in a classroom are prototypically directed by teachers’ guidance and institutional practices (Danby, 2002). Within the terrain of classroom communication, analysts have placed great emphasis on the IRF (i.e., teacher initiation-learner response-teacher feedback) patterns or question and answer adjacency pairs between teacher and students (Mori, 2002). Nevertheless, there are various entries to understand the orderly construction of classroom activities. Interactions and learning do not necessarily unfold around teachers’ knowledge and questions in class (Houen et al., 2018). For instance, student-initiated questions reflect students’ learning orientations and may sequentially alter the course of classroom discourse (Duran and Sert, 2021). When the interactional opportunities are guaranteed, contingent learning opportunities emerge through the natural course of interactions. Hence, the interactive class tends to render a communication-friendly environment for language learners. The key point then goes to the fusion of language learning and using the target language for communication in class.

Despite teachers’ guidance, it is relevant to observe the roles that learners play in completing social actions and learning (Merke, 2016). Concerning the complexity of classroom discourse, analytic focuses have been gradually given to peer interactions. The roles that language experts and novice learners play in the classroom are not stationary (Merke, 2016): peers can also perform as the explainer to construct knowledge collaboratively in class (Jakonen, 2018; Stone, 2019). To flesh out the specific features of peer interaction, researchers aim to demonstrate the roles that peers play in the construction of classroom discourse, and the learning outcomes that yield from peer assistance. Research has shown that various factors including proficiency, perceptions of peers and interactional environment significantly affect learners’ engagement in peer interaction (Dao, 2020). It has been reported the investigation of peer interaction as an observable phenomenon contributes to the reconceptualization of L2 learning (Eskildsen, 2018; Jakonen, 2018). For instance, when students retrospectively orient to learning achievements as a resource for constructing peer interaction, their learning becomes an observable co-constructed action (Jakonen, 2018). In that sense, the detailed analysis of peer talk makes a better understanding of how learning is contingently situated in peer interaction, and how moment-to-moment private talk is managed by learners without assistance from language experts. Thereby, more research should consider learners’ agency, initiatives, and interactional framework in the classroom. In contrast to interactional practices between teachers and students, private interactions within peers in class are infrequently reported. How do learners rationalize public pedagogical tasks in private? How do learners render each other reciprocal interactional space and assist learning while making use of available resources if they do? How do learners mobilize their agency to learn and manage the classroom discourse? These questions are better answered through the detailed analysis of peer interaction.

In addition to the dichotomy of teacher–student interaction and peer interaction in the classroom (Jakonen, 2018), on-task and off-task interactions are distinguished to explicit different interactional trajectories (Illés and Akcan, 2017). Central to this categorization, on-task interaction refers to interactional trajectories within conventional classroom communication; off-task interactions feature the everyday language use in the classroom. Public interactions following pre-designed instructions and executing required tasks are seen as on-task, while interactions that do not proceed with the current tasks are typically analyzed as off-task (Hauser, 2016; Stone, 2019). While the on-task classroom talk prototypically unfolds around teacher-initiated questions and students’ responses (Duran and Sert, 2021), the off-task talk reflects characteristics of real-life interaction (Markee, 2005). When learners perform beyond the conventional interactional sequence in class, the unplanned language use activates both their linguistic and metalinguistic skills (Illés and Akcan, 2017). According to Waring (2012), these moment-by-moment uninvited learner initiatives in class reflect how learners mobilize various resources to manage their learning. Despite the growing studies on on-task interactions, a relative lack of attention has been given to the detailed analysis of off-task peer interactions. The recent exceptions include the studies on L2 learning in the wild (Illés and Akcan, 2017; Eskildsen, 2018), a study about how L1 is used in peer interaction to facilitate L2 learning (Jakonen, 2018), and a report of repair in an off-task talk in a Japan EFL classroom (Stone, 2019). Among these studies, Illés and Akcan (2017) analyzed how off-task interactions in the classroom incidentally link to the language used in the real world and argued for encouraging off-task interactions during the process of L2 learning. Findings from Jakonen’s study (Jakonen, 2018) also suggest that the document of peer interactions and off-task talks in language class facilitates conceptualizing how L2 learning unfolds in interaction.

### Conversation-for-learning and learning opportunities

Under a variety of guises, L2 learning is being seen as a pervasive phenomenon embedded in mundane interactions when L2 learners use the target language to do social actions (Wagner, 2015; Eskildsen, 2018). Opportunities for conversation that are limited in conventional teacher-fronted language classes are currently widely accepted as fundamental for L2 learning (Eskildsen, 2018). Restricted by the requirement to fulfil rigidly prearranged teaching tasks and to realize specific pedagogical goals, interactional opportunities are heavily limited in conventional teacher-fronted language classes (Kasper, 2004; Hauser, 2013; Kasper and Kim, 2015; Illés and Akcan, 2017). Following the call to emphasize the interactional competence of EFL learners (Kasper, 2006; Young, 2008; Barraja-Rohan, 2011), researchers and practitioners argue that language should not be learned in a vacuum. Pedagogical arrangements, therefore, such as language immersion program (Ahlund and Aronsson, 2015), communicative language class (Stone, 2019), language café (Engwall et al., 2021) and conversation-for-learning (Kasper, 2004; Kim, 2012, 2017a,b; Hauser, 2013, 2017a,b; Kasper and Kim, 2015; Zimmerman, 2020) are implemented to augment L2 learners’ exposure to and the employment of the target language, offsetting the limitations of conventional language teaching. Although the titles of these arrangements are diverse, they are indeed homogeneous in terms of rendering learning opportunities for learners and establishing a flexible participation framework. Thus, as Kasper and Kim (2015) have argued, these nonformal institutional talks can all be referred to as conversation-for-learning (CfL). Previous studies have highlighted several distinct features of CfL in contrast to the conventional classroom discourse. First, instead of being perfect language models, language experts perform as conversational partners (Hauser, 2017a,b). In another sense, the significance of the non-gate-keeping interactive activities is to provide additional oral practicing opportunities rather than guaranteeing the learning outcomes. Second, communications at CfL prioritize the content instead of the form (Hauser, 2017a; Kim, 2017b; Engwall et al., 2021). Thereby, as interactions in CfL are primarily communication-driven, language experts frequently refrain from their initiations of repair and corrections for the progressivity of conversations (Hauser, 2017a). Third, reciprocal peer interaction is highly recommended and encouraged (Kasper and Kim, 2015). As CfL highlights the significance of “doing” conversations, learners’ participation and agency are re-emphasized.

The existing literature on conversation-for-learning (CfL) has shed light on how language learning opportunities are contingently generated in naturally occurring interactions when participants are accomplishing social actions (Doehler and Pochon-Berger, 2011; Kim, 2017b). CfL settings arranged out of the classroom are commonly classified as usage-based due to the target language being mainly practiced for reaching mutual understandings. Learning in these settings is naturally generated without prompting tasks and clear pedagogical goals. Thereby, learners’ autonomous awareness and willingness are pivotal for their learning orientations. There are many entries for L2 learners to get access to learning opportunities through conversational practices. For instance, knowledge asymmetries between a language expert and novice learners can generate definition sequences for acquiring new words in the target language (Kim, 2017b). Contingent vocabulary learning opportunities also occur when participants perform negotiation for meaning practices (Eskildsen, 2018). Besides, code-switching and the use of learners’ L1 occasion opportunities for L2 learning (Zimmerman, 2020). These CfL studies typically analyze dyadic interactional practices between an L2 learner and a language expert. The exceptional cases can be found in Kim’s (2017a,b) studies in which the researcher investigated a tripartite CfL arrangement. The analyst reported how two Korean EFL learners benefit from communicating with an American native speaker. Still, aligning with other CfL studies, the aim was to investigate how language gaps and knowledge asymmetries between language experts and learners facilitate L2 learning (Kasper, 2004; Hauser, 2013, 2017a,b).

However, the presence of an L1 conversation partner is not necessary for a CfL setup (Kasper and Kim, 2015). As learners can generate reciprocal learning opportunities for each other, learning also frequently occur in peer talk. The analysis of interactive peer activities is thereby significant for the understanding of autonomous L2 learning. Further, interactional practices are subject to the number of co-participants. It is anticipated that multiparty interactions can be significantly different from those in dyadic or tripartite settings. In particular, the presence of other parties may significantly influence the repair sequence (Forrester, 2008; Bolden, 2009, 2011). However, very few CfL studies report the real happening learning behaviors in the multiparty arrangement within peer groups. Therefore, it is still vague why peer interaction is initiated at some time points, and how learning opportunities are managed within peer interaction. CfL arrangements incorporating multi-participants (more than two learners), thereby, deserve closer analytic observations.

Given classrooms are still the core arena for second language learning *per se* (Wagner, 2015), linking language in nature to classroom teaching is omni-relevant. Thereby, language educators and decision-makers are imitating out-of-class learning environments and moving them into the classroom with fewer limitations on interactional topics in class. Against this backdrop, increasing interactive classes seen as hybrids of usage-based interactions and task-based teaching are executed to guarantee L2 learners’ exposure to the target language. Under the guidance of communicative language teaching, these classes encourage L2 learners to actively participate in conversational practices so that they can practice the use of the target language and enhance their communicative skills (Wong and Waring, 2020). These interactive language arrangements, to some extent, break the conventional patterns of classroom discourse. Without explicit pedagogical designs and teaching goals, these courses prototypically render only implicit interactive topics to create an interaction-friendly environment. Even though these courses try to imitate natural conversations in the real world, interactions in the classroom are significantly different from ordinary talks. Therefore, L2 classroom interaction should be regarded as a variety of institutional discourse (Seedhouse, 2004). In a classroom “under the guise of free conversation” (Kim, 2017b, p. 2), participants make use of their available language resources to imitate real-life language use, while the main interactional trajectories remain in task-based activities. Interactive language class, then, is better to be framed as a distinctive genre: a hybrid of institutional talk and natural conversation.

Different from sheer usage-based settings, interactive class encourages peer interactions and group discussions but still arranges interactional tasks and activities with the necessary supplement of authentic materials (Wong and Waring, 2020). To distinguish previously reported usage-based CfL outside of classrooms, CfL class is better referred to as institutional (Kasper and Kim, 2015) and instructional (Kim, 2017b). In these CfL arrangements, language experts (LEs) offer guidelines for classroom activities and guarantee the occurrence of language use. LEs, thereby, functionalize differently from conventional teachers. The primary duty of LEs is to offer learning opportunities instead of explicit teaching. Briefly put, teachers in teacher-fronted classes occupy the majority of interactional places; LEs in CfL, instead, prompt learners’ use of the target language and provide feedback only when it is necessary. Note that even arranged in the classroom, CfL class is still different from conventional language class for there is no “desired product” being expected (Illés and Akcan, 2017, p. 3).

Different from teacher-fronted teaching, instructional CfL arrangements have a significantly higher tolerance for off-task interaction and peer communication. Therefore, different interactional trajectories should be analyzed to reach a rather comprehensive understanding of the organization of multiparty CfL. Briefly put, participants are expected to observe others’ interactions when it is not their turn to talk; they are assigned more space to initiate private talks when they feel necessary. Staying tuned in public activities and initiating individual interactional topics require learners to mobilize various available resources for sophisticated designs. When several interactional trajectories intertwine, co-participants have to collaborate with each other to maintain the orderly progress of multiparty interaction. Alternatively, interactions in a multiparty classroom may result in chaos.

In sum, more analytic attention should be given to interactive details in multiparty CfL in terms of its organization and learners’ autonomous learning behaviors. A detailed analysis of peer interactions, thereby, facilitates the understanding of how learning opportunities are contingently generated and managed in private. Given the complexity of multiparty interactions in L2 learning arrangements, the question of how different parties mobilize various resources to stay tuned in interactional tasks and maintain intersubjectivity is barely answered. Thereby, examining different interactional trajectories may contribute to understanding how learners rationalize public talk in private and how different participation framework is utilized for learning and orderly construction of multiparty discourse.

### Reconceptualizing learning in CA-for-SLA

One of the basic tenets of CA-for-SLA is language learning is an observable process that happens in social interactions (Markee and Kasper, 2004). By redefining language learning and use as social actions, CA-for-SLA studies “inform the teaching of languages in new and radical ways” (Wagner, 2015, p. 76). Conceptualizing learning in CA-for-SLA, learning L2 is now constructed as a temporally observable action (Sahlström, 2018) and a usage-based process (Eskildsen, 2021). While most SLA research is theory-driven, CA-for-SLA studies discard the researcher-centric view, exploring learning behaviors and processes only through learners’ displays (Markee, 2008). In another sense, CA-for-SLA aims to understand how learning occasions unfolds in real-time interaction by either tracking learning objects or the learning process (Markee, 2008).

Prior CA-for-SLA research has shown that conventional classroom discourse demonstrates a similar pattern. Much analytic attention has been given to teacher-directed speech or the IRF sequence. In particular, learning as happens in repair sequence has been discussed in many CA-for-SLA literature. This strand of studies highlights how learners learn the target language and accumulate knowledge through participating repair sequences between learners and language experts. In contrast, relatively little analytic attention has been given to learning behaviors beyond repair and the IRF sequence. To date, a growing body of CA-for-SLA research aims to deconstruct the organization of classroom discourse by analyzing how interactions among different co-participants unfold in the classroom. The analytic focus is to examine how co-participants utilize different participation frameworks to construct the orderly organization of classroom activities. For instance, a very recent study shows how teachers manage classroom arrangements through discursive practices (Klattenberg, 2021). Similarly, this line of research prototypically examines the interactional process between teachers and students (e.g., Klattenberg, 2021; Van Der Ploeg et al., 2022). Although students’ active participation is acknowledged, the literature on how learners manage their learning remains sparse. Thus, studies that examine learning from learners’ perspectives and emphasize learners’ agency are in need in the terrain of CA-for-SLA.

## Methodology

### Setting

**Figure 1:**
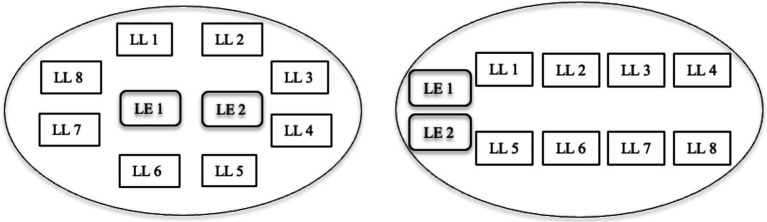
Classroom arrangements.

The research took place in three CfL classrooms at a university in Macau. To meet the language requirement needed for English-medium education, the university arranged a weekly interactive English course to amplify learners’ exposure to and the application of English. In each of the chosen classes, there were two language experts (LEs) and eight language learners (LLs). To create a communication-friendly classroom, students frequently sat in a circle or in two lines (Figure 1). Activities were not rigorously arranged and pre-planned so that more interactional opportunities and space can be offered to students. The underline tenet was learning opportunities will naturally emerge from both classroom activities and interactions.

### Participants and data collection

Data were drawn from 10 h of videotaped CfL classes in which two language experts (LEs) Dan (a native speaker of English) and Miranda (a Chinese proficient L2 user of English) participated. In total, 24 first-year university students with relatively low English proficiency levels (A1–A2 CEFR) aged between 18 and 20 participated in the study. They all spoke Mandarin and/or Cantonese as their first languages. Informed consent forms were obtained from all participants before the recording. Considering the complexity of multiparty interactions, two cameras were set in different corners of the classroom to capture as many non-verbal actions as possible. Additional two audio recorders were placed to assist in recording participants’ verbal productions.

### Data analysis

This research follows the canonical CA methodology (Sacks et al., 1974) that is subject to the construction of social orders through the scrutiny of talk-in-interaction within its most proximal contexts. From the very beginning, empirical CA research relies on the technology of recording to collect data in terms of naturally occurring interactions. Further, the modern utilization of video-recording devices facilitates capturing and preserving both verbal and non-verbal details in real-time interactions. The analytic goals of CA are then extended beyond the sheer examination of talk and language but to the human actions manifested by talk (Seedhouse, 2004). Through analyzing both vocal and non-vocal behavioral performance as interactive resources that interactants display in interaction (Mondada, 2019), CA studies present how social members systematically generate meaning in the process of accomplishing social actions.

As even seemingly trivial details in daily life can be crucial for the orderly construction of social interaction, CA transcripts mark talk-in-interaction in meticulous details and prepare it for retrospective scrutiny. Data analyzed in the present study come from about 10 h of videotaped classes, which were transcribed primarily following the classic transcription conventions developed by Jefferson (2004) with embodied actions and the onset of actions were marked (Mondada, 2018; Greer, 2019; see Appendix A). For ethical concerns, pseudonyms were assigned to participants in the transcripts. To make the recorded data accessible to both analysts and readers, details in recorded interactions including stress, silence, intonation, gaze, and posture were marked meticulously in transcripts. Actions were further taken to guarantee the quality of transcripts to the maximum: as one of the researchers transcribed the data, the other contributed to checking the accuracy.

Sequential analysis of data also followed the CA conventions: the analysis process was data-driven and did not start with any theoretical assumption. After a collection of similar interactional instances was established, the analysts identified patterns that were performed as normative. A total of 42 instances of peer interactions were identified in this 10-h dataset. We then highlighted the occurrences of private peer interactions and explicated how learning opportunities were contingently occasioned by public repair sequences.

## Recycling on-task words in private peer interaction

**Figure 2:**
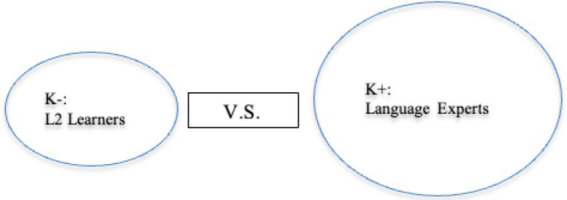
Discrepancy between language learners and language experts on the target language resources.

**Figure 3:**
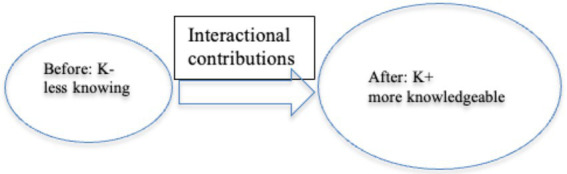
Possible individual epistemic change from a less knowing stance to an updated more knowledgeable condition.

Drawing upon Heritage’s (2012) notion of the epistemic gradient among interactants, language experts are saliently more knowledgeable (K+) than less knowledgeable (K–) L2 learners. However, individuals’ epistemic stances are not invariable: they may alter from moment to moment due to interactional achievements (Heritage, 2012). Then, the conceptualizations of K– and K+ can delineate the knowledge discrepancy between learners and experts or the possible individual epistemic change from less knowing to more knowledgeable. Figures 2, 3 display different referents of K– and K+ employed in the present study.

In classroom contexts, teacher-directed interactions are prototypically prioritized. As learners who do not hold interactional floors are expected to observe classroom activities mutedly, entering such a language arena that is dominated by language experts is both challenging and motivating for L2 learners (Illés and Akcan, 2017). Despite language expert-directed interactions, there are moments when learners spontaneously initiate negotiation for meaning practices in private. Excerpts below display how public interactions are relevant for other participants and how onlooking learners mobilize interactional space in peer interactions for their learning opportunities.

### Efficient peer interactions

Prior to Excerpt 1, language experts ask learners to describe their characters. Before the focal scenario, several learners have given their words such as shy, outgoing, and hardworking promptly, and it now comes to Francis’ turn.

*Excerpt 1. pathetic*


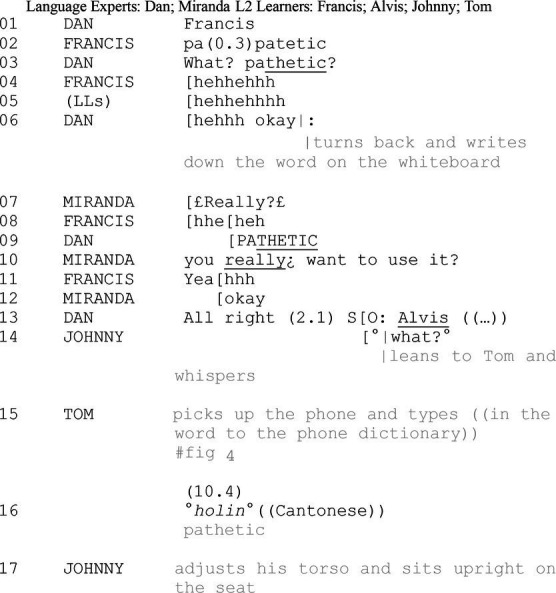


Dan (LE) assigns the turn to Francis (LL) by summoning his name (line 01). Francis renders the word “*patetic*” (line 02). He initially pronounces the first syllable “*pa*” and stops for a 0.3-s before he self-repairs the word to “*patetic*,” revealing his slight unsureness and difficulty in pronouncing (line 02). Dan prompts ratification and subsequently recasts the problematic word as “*pathetic*” in the same turn (line 03). Given it is somewhat unexpected to hear people depicting himself as pathetic, both LEs (Dan and Miranda) initiate repair attempts (respectively in line 03, line 07, and line 10) to offer Francis interactional floors to either ratify or reject the proposal of “*pathetic*.” After three attempts to confirm with Francis (line 04, 08 and 11), both LEs acknowledge the word: Dan formally writes down the word on the whiteboard (06); Miranda registers with “*okay*” (12).

**Figure 4:**
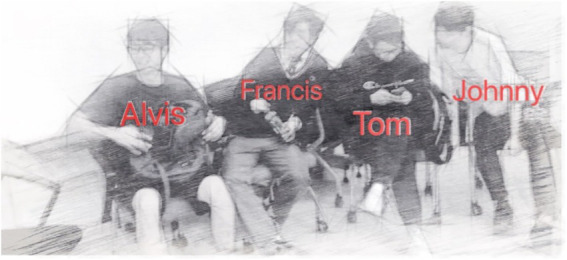
Frozen action: looking at the phone screen.

“*All right*” as a concluding mark, the sequential sizable 2.1-s pause, and a “*so*” in the same turn (13) display Dan’s sophisticated design to close the current sequence and his tendency to advance tasks with another recipient (Alvis). Overlapping with the prolonged “*so*,” nevertheless, a third party learner (Johnny) in the audience initiates a private “*what*” in line 14 to his peer (Tom) sitting next to him, onsetting a personal peer behavior (Hellermann and Cole, 2009). The abrupt initiation of “*what*” disjoints the main “immediately preceding talk” (Schegloff, 2000, p. 207). As an open-initiator for repair, “*what*” does not target or locate any specific repairable item or component in the proceeding sequence (Drew, 1997). Tom’s prompt action of picking up his phone, however, demonstrates his analysis of the prospective trouble source. The quick action of resorting to a dictionary indicates Tom’s K– knowledge with “*pathetic*” and demonstrates his orientation to update his stance to K+. Consider their positions as in Figure 4: Johnny is leaning on Tom, looking at Tom’s phone screen. The change in Johnny’s body position demonstrates his heightened concentration. Occupying a lengthy 10.4-s pause, both Tom and Johnny silently stare at Tom’s phone screen. The statuesque frozen action reveals both participants regard the dictionary as the authority (Norris, 2020).

Instead of resorting to salient K+ participants (Dan, Miranda, and Francis), Johnny (K–) initiate private peer talk to address his understanding difficulties. He cooperates with Tom (K–) to solve their problems locally within intimate peer interactions. Now consider the below Excerpt 2.

*Excerpt 2. ruins*


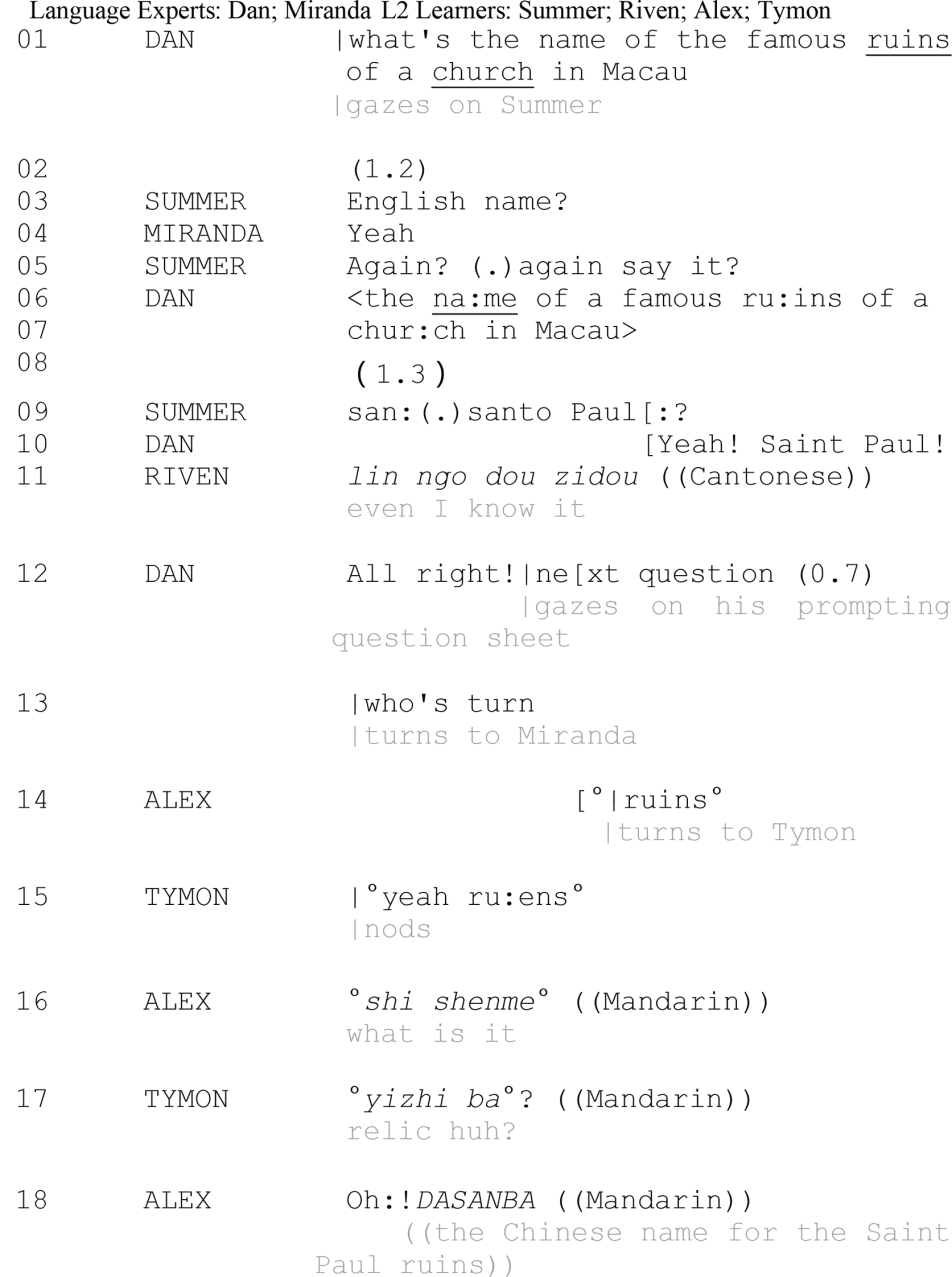


In line 01, Dan issues the question and selects Summer as the recipient. Given the analytic focus of the study is on peer interactions, detailed analysis of LE–LL interactions from line 01 to 13 is excluded. Starting from line 14, Alex selects his peer (Tymon), initiating a private interactional trajectory. The onset of the private talk overlaps with but does not interrupt the public interactional task. Alex retrospectively targets the word “*ruins*” embedded in the proceeding repair sequence and resorts to a third party (Tymon) to negotiate for the meaning. The repetition of “*ruins”* (line 14) fails to specific his designs to Tymon, who reiterates “*ruins”* as his receipt (line 15; Greer et al., 2009). Sequentially, Alex builds on Tymon’s turn and explicates his question by asking what it is (line 16). Tymon renders an L1 referent with an uncertain question maker “*ba*” (line 17). The subsequent occurrence of elongated “*Oh*:” (line 18) as a change-of-state token (Heritage, 2010) registers Alex’s information receipt, indicating that Alex’s epistemic stance has been updated: alter from “not-knowing to knowing” (Sidnell, 2009, p. 105).

Instead of resorting to interlocutors in salient K+ stances, Tymon tackles his understanding problem with peers. Excerpt 2 demonstrates how public repair occasions unprojected learning opportunities for onlooking participators. It presents how the seemingly peripheral learner draws himself and a peer into a local learning opportunity by initiating private peer interactions and changing their participation role from the onlooking audience to active interlocutors. It shows peer interaction facilitates the learner to acquire the meaning of the focal word and retrospectively comprehend the initial question in the beginning line. Alex, in line 18, eventually renders a Chinese referent for the question Dan inquiries in line 01.

### Inefficient peer interaction

The analysis in this section delineates an instance in which learning opportunities are initially oriented to but are sequentially abandoned in private. It shows how the private interaction closes without a notable agreement and salient learning outcomes.

*Excerpt 3. enthusiasm*


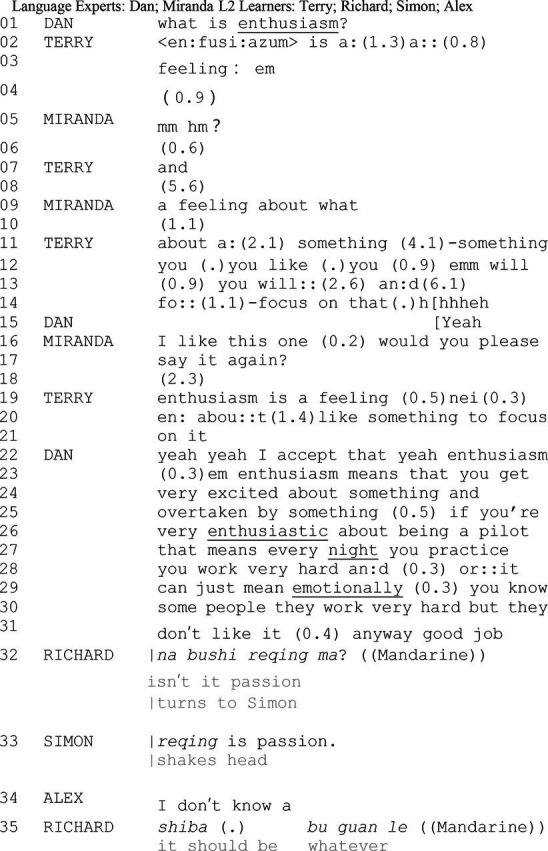


Dan initiates a public talk about “*enthusiasm*” in the beginning line with Terry. While Terry attempts to explain “*enthusiasm*” (line 02–04, line 07, line 11–14, and line 19–21), Dan and Miranda scaffold Terry’s utterance. Now and then from line 05–17. From lines 22–31, Dan clarifies the meaning of “*enthusiasm*” to scaffold learners’ interpretation. It is quite salient here; Dan closes the talk around “*enthusiasm*” by “*anyway good job*” (line 31). In the most immediate next turn, Ricard (LL) initiates a disjunctive question to his peer (line 32). Given that both “*enthusiasm*” and “*passion*” are frequently translated to “*reqing*” in Chinese, the initiation endorses Richard’s orientation to a rather vague understanding of “*enthusiasm*” and his attempt of providing a candidate understanding of enthusiasm as passion. In contrast to the *wh*-question, disjunctive questions convey an assumption to a larger extent by offering a candidate understanding (Svennevig, 2008). In another sense, Richard seeks to confirm his understanding of “*enthusiasm* as “*reqing*” from his peer. However, the proposal of referring “*enthusiasm*” to “*passion*” is sequentially rejected by the selected peer Simon (line 33). Shaking his head and linking what Richard has uttered (line 32) to a synonym “*passion*,” Simon rejects Richard’s proposal of equalizing enthusiasm as “*reqing*” or “passion.” The disaligning discussions of enthusiasm, further, draw another learner (Alex) into the private interaction. Alex self-reports his stance toward the negotiating sequences by clearly uttering an “*I do not know*” (line 34). There may be different interpretations of Alex’s account. One may agree that either he confuses about the meaning of the focal word, or he intends not to be involved in possible further discussion about the word. In either case, Alex’s reaction fails to support the progressivity of the private interactional task. Richard, then, self-closes the private interaction he initiated with a “*forget it*” and a “*buguanle* (whatever)” (line 35) which indicates that the peer interaction around “*enthusiasm*” is abandoned. Notably, before he concludes their negotiation, Richard repeats his stance, which still sticks to his original understanding of the word. The “*shi ba* (it should be)” in line 35 conveys that Richard adheres to his initial understanding of the focal word. His peers, both Simon and Alex, however, fail to advance each other’s understanding in the private negotiation-for-meaning sequence. In this episode, participants’ knowledge and understanding are not saliently updated to a K+ stance. Albeit peer interaction offers temporal learning opportunities, it yields only invalidate learning outcomes. This peer interaction is then classified as inefficient in terms of learning outcomes. Nevertheless, it still presents how a temporal learning opportunity is managed in private peer interactions.

## Concluding discussion

Drawing on CA-for-SLA, this study has analyzed instances that showcase how private learning practices are contingently occasioned in CfL class and how learners manage learning opportunities within peer interactions. Reported instances in the study present how public repair practices elicit private negotiation for meaning practices in peer interactions. What is clear is that the extracts presented above involve learner-initiated private interactions that build on public repair practices. Learners repeatedly retrieve focal trouble words embedded in prior repair sequences for their private learning opportunities. The detailed analysis has demonstrated how the on-task repair is extended, managed, and completed in private peer interactions without language experts’ guidance. L2 learners orient to their peers to tackle understanding problems and learning opportunities. Through peer interactions, learners make the knowledge gaps that they identify from on-task activities relevant for individual learning. The process changes their participation framework from the onlooking audience to an active learner, revealing how L2 learners maximize their agency to locate an interactional niche for private learning opportunities.

This study extends prior work on learning orientations and behaviors in multiparty CfL arrangements. Instead of resorting to language experts, learners make use of the inconspicuous knowledge asymmetries within the peer group for their private learning opportunities. Aligning with Kim’s (2017b) observation, learners demonstrate a preference for vocabulary learning. In contrast to the previous literature about the role that K+ language experts play in scaffolding learning (Hauser, 2017a,b; Kim, 2017a,b), the study highlights the occurrences and features of peer interaction in multiparty CfL. It presents peer interaction as a dynamic process in which co-participants exhibit their agencies to manage their learning. Thus, it highlights the importance of extending the analysis of L2 learning behaviors to peer interactions. It also demonstrates how different interactional trajectories simultaneously unfold in multiparty CfL settings for L2 learning.

The co-existence of multiple interactional trajectories addresses the rather under-reported schisming (Egbert, 1997) phenomenon in the classroom. The finding of this study shows that L2 learners display their sense-making of public on-task talk within an immediately sequential PRNfM sequence. When the on-task repair triggers understanding problems, onlooking participants request peers’ extra information and assistance to remedy (Svennevig, 2008). The delicate beginning point of peer interaction indicates L2 learners’ efforts to balance the orderly construction of classroom discourse and their orientations for learning. Extracts presented show that learners initiate peer interactions at a time point when the learning opportunity is being abandoned by others. In addition, initiators of peer talk intend to close private interactions in minimal turns so that the private interactional trajectories rejoin the public class activities promptly. The rather short but multi-faceted PRNfM sequences endorse learners’ efforts to sustain the overall orderly construction of the classroom. For instance, the initiator of the peer talk, in Excerpt 3, self-closes the private sequence without yielding efficient outcomes. Instead of being interruptive and distracting, peer interaction frequently facilitates the orderly progress of classroom activities. The argument is also supported by Except 2 in which the focal learner makes use of the information he acquires from his peer in the PRNfM sequence to understand the prior public task.

**Figure 5:**
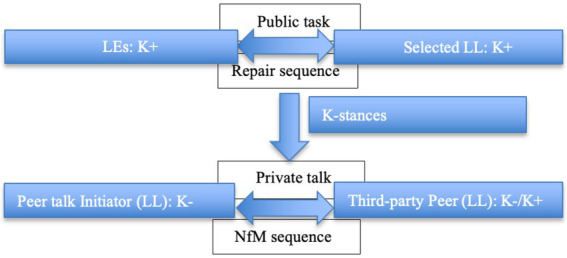
Relevance of the public repair sequence for private peer talk.

Pertaining to the changing participation framework, the study showcases how seemingly peripheral learners in the preceding interactional tasks attend to the contingent learning opportunities by co-constructing peer interaction. Selected excerpts to display the relevance of public repair for the peripheral audience: the public repair between assigned learners and LEs occasions other (the third party)-initiated-another (the third party)-repair sequence in private peer talk. Different from the widely reported other-initiated-other-repair practice, Forrester (2008) refers to the aforementioned interactional practice as “other-other repair,” in which a third party is an initiator and another third party acts as the repairer. Figure 5 depicts how the public repair sequence is made relevant for the private learning opportunities.

Although hearers’ epistemic stance is highly relevant to the speaker (Heritage, 2012), it is almost impossible for speakers to check upon every participant’s understanding from moment to moment in a multiparty context. The analysis showcases the relevance of public repair for the onlookers’ sense-making (Bolden, 2009, 2011). As public repair highlights knowledge asymmetries, onlooking learners self-check their epistemic status. When onlooking learners identify themselves in a K- stance, they do not instantly compete for the interactional floor or interrupt the proceeding public on-task talk. Instead, they wait until the sequence is closing and there are salient signs for the unfolding of a new sequence. The entry points of private peer interactions endorse the audience’s efforts to sustain the orderly organization of the current interactional tasks and their sophisticated designs for the most imminent time point to orient to learning opportunities. Thereby, PRNfM sequence as an intersection between public talk and private learning reveals how learners rationalize their learning opportunities in private. By lowering the volume and selecting recipients from the onlooking audience, participants simultaneously prioritize on-task talk and initiate a private interactional trajectory. Thus, initiators of peer interaction set up a new private participation framework for themselves and designated peers, which alters the participating role from a silent onlooker to an active interlocutor and learner.

By presenting the features of underlying peer interaction, the current study reveals the complexity of classroom discourse by showcasing the co-existence of multiple simultaneous interactional trajectories. That is, several diverse interactional trajectories intertwine each other. When public classroom activities occasion private interactional trajectories, peer interaction presents its close relevance to the on-task talk. The study, thereby, rejects conceptualizing the tacit moment-by-moment co-constructed peer interaction as off-task. In contrast to the rather simplified dichotomy of classroom discourse as on-task and off-task as documented in Illés and Akcan’s (2017) and Stone’s (2019) research, the study shows how the seemingly “off-task” peer interaction is indeed closely attached to primary classroom activities and facilitates learners’ autonomous learning. To summarize, extended repair in private peer interaction constitutes a venue for understanding the orderly construction of multiparty classroom interactions.

While Jakonen (2018) acknowledges the retrospective practices for learning in separated peer talk, the present study highlights occasions of vocabulary learning in the most proximal PRNfM sequence in private. Thereby, peer interaction as a multi-layered activity requires more analytic attention. While student-initiated questions reflect their learning orientation (Duran and Sert, 2021), peers’ responses to these questions demonstrate how learners comprehend and process each other’s learning opportunities. As learners demonstrate a similar pattern to initiate private learning-oriented sequences upon the completion of the public repair, this small collection of instances also contributes to explaining the very central “why that now” question in CfL classes. While public repair offers salient learning opportunities, repair that is not adequate for participants’ sense-making elicits private peer interactions.

Methodologically, the research supports other CA-for-SLA studies by presenting how the employment of CA makes the temporality of learning observable in the learning process. As CA explicates the process of constructing shared knowledge, it provides researchers with an analytic tool to understand the construction of an interactive language classroom. Instead of focusing on learning outcomes, CA-for-SLA tracks the dynamic process of learning. In another sense, through the lens of CA, the study presents L2 learning as a dynamic, temporally observable, and co-constructed process. It reveals the minute ways in which the order of classroom discourse is managed. Although co-participants utilize different participation frameworks for individual interactive agendas, they closely observe the progress of other interactional trajectories.

Despite pedagogical and methodological implications, the study has its limitations. First, due to the limitations of recording devices, some interactional moments of the private talk were unclear to the analysts. That is, even if the cameras captured participants’ non-verbal movements, the audio quality was, to some extent, disappointing. The research then had to exclude those excerpts from the analysis. It would be helpful for future research to use wearable cameras and microphones for better audial and visual quality. Second, further research may consider longitudinal learning achievements through tracking learning objectives. As the present study reports only temporal learning orientations, it would be significant for other studies to examine whether the learning outcomes yielded from peer interaction are sustained over time.

## Data availability statement

The original contributions presented in the study are included in the article/supplementary material, further inquiries can be directed to the corresponding author.

## Ethics statement

Ethical review and approval was not required for the study on human participants in accordance with the local legislation and institutional requirements. The participants provided their written informed consent to participate in this study. Written informed consent was obtained from the individual(s) for the publication of any potentially identifiable images or data included in this article.

## Author contributions

MC: conceptualization, methodology, data collection, data analysis, and writing—original draft and revision. SY: data analysis, visualization, and writing—revision and editing. All authors contributed to the article and approved the submitted version.

## Conflict of interest

The authors declare that the research was conducted in the absence of any commercial or financial relationships that could be construed as a potential conflict of interest.

## Publisher’s note

All claims expressed in this article are solely those of the authors and do not necessarily represent those of their affiliated organizations, or those of the publisher, the editors and the reviewers. Any product that may be evaluated in this article, or claim that may be made by its manufacturer, is not guaranteed or endorsed by the publisher.

## Appendix A

### Transcription conventions

Transcriptions are primarily based on Jefferson (2004) with minor adaptions.

**Table 1:**

nods	embodied actions in unnumbered sub-tier in gray font.
*reqing*	Chinese words transcribed in italics.
|	locates the onset of the action.
#fig	the exact moment at which a screenshot has been taken.
